# Age-related decrease of odorant sensitivity for a selection of nine diverse molecules

**DOI:** 10.1007/s00405-025-09254-7

**Published:** 2025-02-24

**Authors:** Agnieszka Sabiniewicz, Malaika Krause, Thomas Hummel

**Affiliations:** Smell and Taste Clinic, Department of Otorhinolaryngology, TU Dresden, Dresden, Germany

**Keywords:** Aging, Olfaction, Odor threshold

## Abstract

**Purpose:**

The ability to smell is known to decrease with age. Still, several studies demonstrate that age-related deterioration is not the inevitable fate of each individual but rather depends on “successful aging” and odorant properties. Based on these notions, in the present prospective study, we aimed to investigate odor thresholds in younger and older adults in response to an extended list of various odorants.

**Methods:**

In 58 participants (31% ≥ 50 years old), we assessed thresholds for nine odorants.

**Results:**

General Linear Mixed Model (GLMM) revealed that, independently of the odor context, younger adults had higher odor sensitivity compared to older ones. Furthermore, while molecular weight did not differentiate thresholds of older and younger adults, it impacted odor thresholds regardless of age, with higher sensitivity noted for lightweight molecules.

**Conclusion:**

In conclusion, based on the present findings, odor thresholds decrease with age, with a pronounced effect for molecules with higher weight. Previous studies suggest that the decline in olfactory function is more significant for certain molecules. However, the current results indicate that this may be an odorant-specific phenomenon, as we did not observe this behavior in our study, which included a selection of nine diverse molecules.

## Introduction

For more than one hundred years, the ability to smell has been known to decrease with age. The findings of Vaschide [1], who showed that aging is accompanied by a decrease in intranasal chemosensory sensitivity to camphor, were later confirmed and extended by numerous modern studies [see for review: 2–4]. The general conclusion remained that the natural aging process typically results in a decline in olfaction, with one in four individuals above 52 years of age exhibiting olfactory loss [5–6]. The prevalence of olfactory dysfunction increases from around 10% at the age of 60 to around 60% at the age of 90 [7–9]. When measured on a 48-point scale using the TDI score (threshold-discrimination-identification), the smelling ability score decreases by an average of 1 point every five years of age [10].

Among olfactory functions, namely odor threshold, discrimination, and identification, threshold appears to be most affected by age [11–12]. Since it reflects odor sensitivity [13], the odor threshold can be considered a base for other olfactory functions [13–14]. While performing the threshold task, participants are presented with diverse concentrations of a given odor to determine the minimum level at which an odor is detected [15].

Why is the sense of smell so impacted by aging? The exact answer to this question remains open [e.g., 16–17] since aging of the olfactory system presumably is complex and involves all kinds of changes, from anatomical to molecular ones. It is also influenced by genetic and environmental factors affecting the olfactory system and the higher centers of information processing [18]. For example, neurogenic changes in the olfactory bulb and olfactory epithelium may cause age-related changes in olfactory function [19], but also early pathogenic exposure may further contribute to the degeneration of the sense of smell over time [15; 17] and even early life experiences may have detrimental effects on sensory function [3].

Given all the above, age-related deterioration of the olfactory function appears to be the inevitable fate of each individual. However, a handful of studies demonstrate the opposite [20–26]. Mackay-Sim and colleagues [27] reported a relatively small impairment in the olfactory abilities of older adults who were in good health, that is, those who did not require medication, did not smoke, and did not have a history of nasal problems (age range: 60–79 years old). By contrast, there was a marked impairment in the olfactory abilities of older persons who did not meet these criteria. Odor properties are another factor that may contribute to successful odor detection in older age. When investigating odor thresholds in response to various molecular weights, Sinding and colleagues [28] reported that older adults were less sensitive to heavy molecules, suggesting they present a heterogeneous olfactory loss that is more specific to heavier molecules.

Based on these notions and our previous work [26] that indicated odor-dependent differences in odor threshold among older and younger adults, in the present study, we aimed to investigate odor thresholds in younger and older adults in response to an extended list of various odors.

## Materials and methods

### Participants

A total of 70 participants (24 men) aged 18 to 71 (mean ± standard deviation 40.9 ± 17 years) took part in the prospective study. Since the study aimed to compare odor threshold performance in younger and older people, 39% of participants in the initial sample were 50 years old or older [29]. The participants completed a questionnaire to obtain demographic information and information on their health status.

Among the most common disorders, allergies (37%) and asthma (7%) were reported. All of these disorders were well-controlled and did not produce complaints at the time of testing. Additionally, one woman who declared nasal polyposis (woman, 27 y.o.) was excluded from the analyses.

None of the participants smoked. Exclusion criteria were: Parkinson’s disease, acute and chronic rhinosinusitis, severe chronic renal dysfunction, and other disorders that would be likely to affect olfactory function like myasthenia gravis; intake of medication likely to affect the sense of smell like chemotherapy.

The appointment lasted about 3 h. The study was performed according to the principles of the Declaration of Helsinki on biomedical research involving human subjects. It was approved by the Ethics Committee at the Medical Faculty of the TU Dresden (application number BO-EK-79022020).

### Odorants

Table 1 presents the 9 odorants employed in the two sessions and chosen due to their different molecular weights and perceptual characteristics.

**Table 1:** Odorants employed in odor threshold testing

Session 1			
odor number	odorant	MW (g/mol)	quality
1	3-methylbutanal	122.16	apple-like
2	Methional	74.12	meaty
3	Damascenone	154.25	honey-like
4	3-hexenal	68.14	grassy
5	Furaneol	140.14	strawberry
6	acetic acid	128.17	vinegar-like
7	Sotolon	192.3	caramel-like
8	ethyl butanoate	132.16	pineapple-like
9	2,3-butanedione	285.34	chlorine-like

### Olfactory testing

We measured odor identification and threshold. Odor identification testing consisted of 16 common odors, with scores ranging between 0 and 16. If the identification score was 12 or higher, the participant was regarded as normosmic [12]. As a result, seven men (54, 59, 54, 56, 66, 69, 53 y.o.) and four women (60, 67, 47, 22 y.o.) had to be excluded because of hyposmia. The final sample consisted of 58 participants (20 men) aged from 18 to 71 years old (M = 38.6, SD = 16.5).

During the threshold test, participants were presented with 16 triplets of pens and required to discriminate the pen containing an odorous solution from two blanks filled with the solvent. Sixteen concentrations were produced by stepwise dilutions in a ratio of 1:2, with 4% odor solution as the highest concentration. Starting with the lowest odor concentration (almost odorless), a staircase paradigm was used, where two subsequent correct identifications of the odorous pen or one incorrect answer marked a so-called turning point. Each turning point resulted in a decrease or increase of the concentration in the next triplet. The threshold score was determined as the mean of the last four turning points in the staircase, with the final score ranging between 1 and 16 points [12].

Except for the standardized odor threshold test, as measured with Sniffin’ Sticks [30], odor threshold was tested for nine odors for two different age groups (Table 1). The testing procedure was identical to the one described above. Nine odorants (Table 1) were consecutively presented to the participants. The odorants were diluted in propylene glycol in a ratio of 1:4, starting from a 4% concentration as the highest concentration. The 8 odor concentrations were presented in glass jars (brown glass, 50 ml volume, opening diameter 45 mm).

Additionally, suprathreshold measures were taken. Each of the nine odors was rated on a scale of 0–10 in terms of intensity and pleasantness, where higher scores corresponded with higher values of the given characteristic (Supplementary Table 1).

Statistical Analyses.

To investigate whether there are systematic differences in odor threshold across age groups in various odor conditions, the final sample was divided into two groups: younger adults (*n* = 40; 69%) and older adults (*n* = 18; 31%). Detailed characteristics of both groups are presented in Table 2. General Linear Mixed Model (GLMM) was run, with odor threshold as a dependent variable and age group (0 vs. 1) together with odor condition (9; see Table 1) as fixed effects variables. Participants’ ID was inserted as a random effects grouping factor.

To further investigate whether odor thresholds for both age groups would differ for variable odors with different perceptual characteristics and molecular weight, we ran a second model where odor ratings (intensity and pleasantness) were inserted into fixed effects variables. Lastly, in the third model, molecular odors’ weight categorized as light (< 126.2 g/mol; 33%), medium (> 126.2 and < 144.8 g/mol; 33%) and heavy (> 144.8 g/mol; 33%) was added to the age group as a fixed effect variable.

For each model, Akaike information criterion (AIC) was obtained. AIC is a single number score that can be used to determine which of multiple models is most likely to be the best model for a given dataset. The desired result is to find the lowest possible AIC, which indicates the best balance of model fit with generalizability.

Data are presented as mean values (± standard deviation). Statistical analyses were performed using JASP v. 0.17 (www.jasp-stats.org), with *p* <.05 set as the level of significance.

## Results

### Descriptive statistics

Both odor identification (M = 13.2, SD = 1.9) and threshold (M = 7.3, SD = 2.7) were measured. Detailed data for the final sample are presented in Table 2, separately for younger and older adults.

**Table 2:** Detailed descriptive characteristics of the final sample, younger and older adults separately

	Participants			
	Younger adults (*n* = 40)		Older adults (*n* = 18)	
	M	SD	M	SD
age	28.9	9	59.8	5.3
Odor threshold	8.9	1.7	5.3	2.2
Odor identification	14.2	1.1	13.2	0.9
gender	men = 30%		men = 44.4%	

Odor threshold scores for singular odorants for both groups separately are presented in Table 3.

**Table 3:** Detailed results of the odor threshold testing for younger and older adults separately

	Younger adults (*n* = 40)		Older adults (*n* = 18)	
	threshold score		threshold score	
odorant	M	SD	M	SD
3-methylbutanal	6.8	1.2	6.5	1.1
methional	7.6	0.5	7.1	0.9
damascenone	6	1.4	5.4	1.3
3-hexenal	6	1.1	5.3	1.2
furaneol	6.7	1.5	5.9	1
acetic acid	6.5	1	5.5	1.2
sotolon	6.4	1.4	5.2	1.1
ethyl butanoate	6.7	0.9	6	1.1
2,3-butanedione	6.4	1	5.4	0.9

### Odor thresholds

**Fig. 1:**
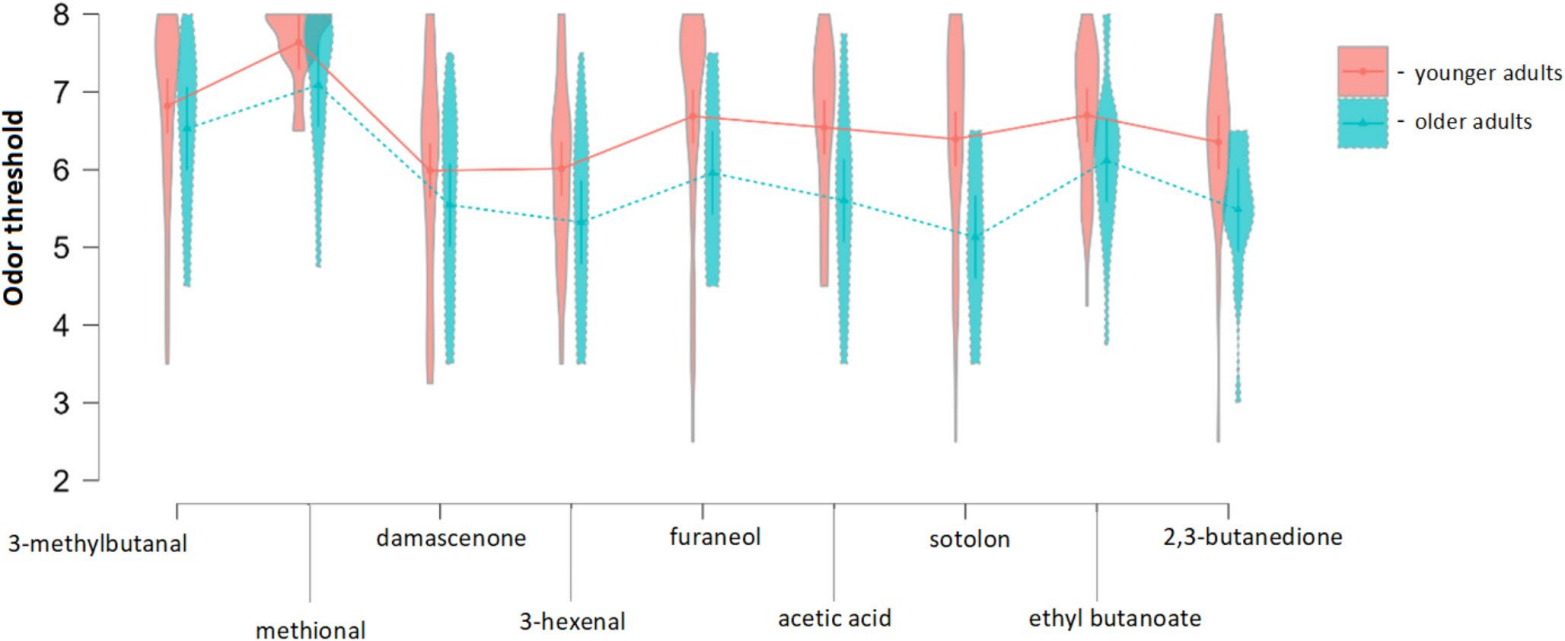
Odor threshold performance in 9 different odor conditions in younger and older adults, respectively. *Note*: Higher threshold scores mean higher sensitivity

The first GLMM indicated that odor conditions did not differentiate between olfactory thresholds in younger and older adult groups (x2 = 9.2, *p* =.329; Fig. 1).

Independently on the odor context, younger adults had higher odor sensitivity compared to the older ones (x2 = 11.9, *p* <.001).

### Perceived odor pleasantness and intensity

Second GLMM showed no interaction between perceived odor pleasantness (x2 = 0.02, *p* =.902) or intensity (x2 = 0.124, *p* <.724) and age groups. An increase in the rated intensity was accompanied by an increase in odor thresholds (x2 = 6.1, *p* =.013).

### Molecular weight of odors

**Fig. 2:**
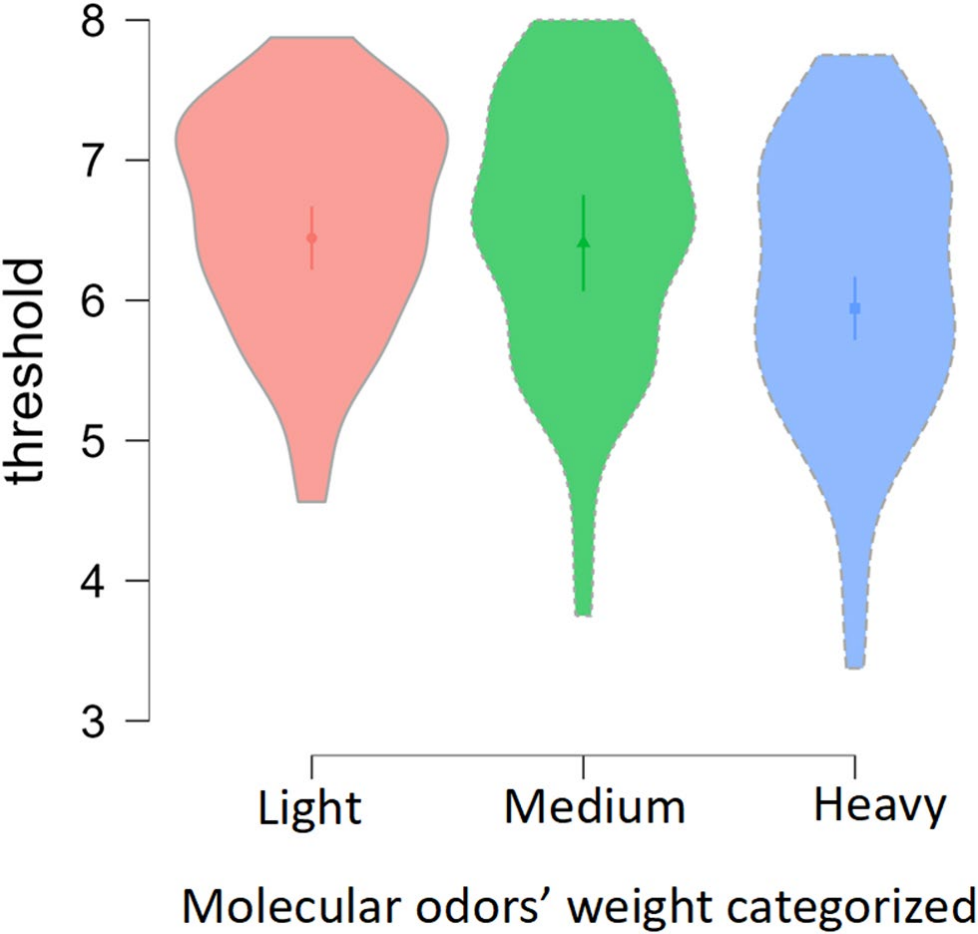
Categorized molecular weight of the odors used in the present study perceived at certain threshold by younger versus older adults. *Note*: Higher threshold scores mean higher sensitivity

Third GLMM demonstrated that molecular weight did not differentiate thresholds of older and younger adults (x2 = 1.1, *p* =.566). Instead, molecular odor weight impacted odor thresholds regardless of age; higher sensitivity was noted for lightweight molecules (x2 = 23.2, *p* <.001) Fig. 2.

## Discussion

Aging of the olfactory system is a complex mechanism that, in most cases, results in a decrease in olfactory function. Still, a growing number of studies indicates that such a deterioration does not affect each individual, and for some odors, the decline is less pronounced or even absent [20–25; 27–28]. Here, we investigated whether odor thresholds in groups of younger and older adults would differ in various odor conditions. Contrary to our hypotheses, the present findings indicate that, regardless of odor condition, younger adults outperformed older ones in the threshold task. As mentioned before, the majority of research in this field demonstrates that aging impacts olfactory function, odor threshold included [e.g., 2–4; 31–32). From an anatomical perspective, the loss of smell function is related to a deterioration of fibers in the olfactory bulb and a decrease in the surface area of the olfactory epithelium [33]. An increase in receptor cell death and a reduction in receptor cell regeneration have also been reported [34–36]. An early decline in olfactory function has been observed in neurodegenerative diseases like Parkinson’s or Alzheimer’s, which may occur before clinical symptoms appear [36]. The reduction in odor sensitivity is gradual and may go unnoticed for an extended period [37].

Even though the present results align with most studies on olfaction and aging, they contradict our primary assumption and previous outcome [26]. Our previous work showed that odor thresholds in younger and older adults changed with different odor conditions, presumably due to the common exposure to some of the odors, their trigeminality, and lipophilicity. More precisely, younger adults outperformed older ones in the majority of presented odors. Still, when eucalyptol and pinene-alpha were used to measure the threshold, older adults performed better than younger ones. Similar findings confirming that age-related changes in olfactory function are not universal have been reported before [20–25; 27–28].

The question remains open as to why, in the present study, we found no odor-dependent differences between younger and older adults. One plausible explanation is that odor sensitivity appears very specific for certain odors. Namely, in our previous work, the odors where people had a high specificity were eucalyptol and pinene-alpha [26], while Sinding and colleagues found differences between age groups for molecules with low molecular weight [28]. Also, the rates of specific anosmia were reported to be higher for heavy molecules than for light ones [38]. Further studies on olfactory function in various groups with olfactory deficits also showed that odor sensitivity is odor-dependent. Parosmic patients with olfactory loss were able to smell odorous triggers of parosmia (coffee and cucumber) on a comparable level to healthy controls. At the same time, this effect was not noticed for phenyl ethyl alcohol [39].

Moreover, due to the limitations of the present study, we cannot exclude that the current sample of older adults was homogenous in terms of physical health [27], depression [40], or cognitive function [41], and hence, no differences in odor thresholds were found. Future studies should investigate this issue further by recruiting numerous and various (successfully and not-successfully-aged) participants. Lastly, since the sample size was not large enough, we could not correct for potential confounders like the presence of asthma or allergies, which should be taken into careful consideration by further studies.

Overall, the odor threshold decreases with age, and this is dependent on molecular weight. Considering other studies, the decrease in olfactory function is more pronounced for some specific molecules. However, this seems to be a specific phenomenon because we have not observed such behaviour in the present study for a selection of nine diverse molecules.
